# The Role of 68Ga-Pentixafor and 18F-Fluorodeoxyglucose (18F-FDG) PET/CT in a Case of Multiple Myeloma

**DOI:** 10.7759/cureus.101195

**Published:** 2026-01-09

**Authors:** Abhishek Kumar, Tamojit Chaudhuri, Manisha Pradhan, Purshottam Singh, Binay Gupta

**Affiliations:** 1 Nuclear Medicine, Tata Main Hospital, Jamshedpur, IND; 2 Medical Oncology, Meherbai Tata Memorial Hospital, Jamshedpur, IND; 3 Nuclear Medicine, Meherbai Tata Memorial Hospital, Jamshedpur, IND

**Keywords:** 18f-fdg pet/ct, 68ga - pentixafor pet/ct, diagnosis of multiple myeloma, multiple myeloma, staging of multiple myeloma

## Abstract

Multiple myeloma (MM) is a type of blood cancer affecting plasma cells in the bone marrow. The diagnosis of MM involves blood and urine tests to demonstrate monoclonal proteins. Imaging modalities such as MRI combined with positron emission tomography (PET) are used for the detection and staging of MM. 18F-fluorodeoxyglucose (18F-FDG) has been used extensively for the staging of multiple myeloma. Another PET imaging modality targeting C-X-C chemokine receptor type 4 (CXCR4) has been recently evaluated for the staging of MM. CXCR4 is a protein receptor expressed on plasma cells, and the radiolabelled Pentixafor radiotracer targets these receptors and is therefore used in PET imaging. In this case report, we compare FDG and CXCR4 PET radiotracers for staging MM.

## Introduction

Multiple myeloma (MM) is a B-cell malignancy characterized by neoplastic proliferation of abnormal plasma cells that secrete monoclonal immunoglobulins [1]. The uncontrolled proliferation of these malignant plasma cells causes bone marrow infiltration and can result in bone lesions, renal damage, and immunodeficiency. International Staging System (ISS) and the Durie-Salmon plus staging (DSPS) system are used for the staging and prognostication of MM. The ISS is a laboratory investigation-based, simple staging system that uses only serum β2-microglobulin and albumin levels and stratifies patients into three stages. Imaging modalities, positron emission tomography/CT (PET/CT) in conjunction with MRI, are also used for the detection of lesions and staging of MM. The DSPS system incorporates the usage of MRI and 18F-fluorodeoxyglucose (18F-FDG) PET/CT for staging of MM based on the total number of lesions [2].

These imaging modalities are also beneficial in the detection of extramedullary involvement and serve as tools for treatment response assessment. A new PET radiotracer, Pentixafor, has recently been evaluated for the staging of multiple myeloma (MM). It targets the C-X-C chemokine receptor type 4 (CXCR4), which is expressed on MM cells. CXCR4 is a transmembrane chemokine receptor involved in tumor growth, metastasis, and the homing of hematopoietic stem or progenitor cells to hematopoietic sites [3]. CXCR4 is overexpressed in multiple solid and hematologic malignancies, including MM [4]. Pentixafor is radiolabeled at a PET/CT facility using the generator-produced radioisotope gallium-68 from a 68Ge/68Ga system and is then used as a PET/CT radiotracer [5]. 68Ga-Pentixafor is an emerging radiotracer with potential applications in MM, certain lymphomas, and possibly improved detection of primary aldosteronism [6]. In contrast to 18F-FDG, this gallium-68-based radiotracer is not dependent on the patient’s blood glucose level, which allows PET/CT imaging to be performed independently of glucose status [7].

The treatment modalities for MM include steroids, chemotherapy, targeted therapies, and stem cell transplantation. However, despite the availability of multiple treatment options, these patients eventually experience relapse or become refractory to therapy. The prognosis for patients with MM remains poor, with a five-year survival rate of around 45.0% [8]. The objective of this case report is to assess the utility of 68Ga-Pentixafor PET/CT in patients with MM and to compare it with the established imaging modality, 18F-FDG PET/CT.

## Case presentation

The patient was a 59-year-old male presenting with complaints of back pain, weakness, and fatigue for three months. He had been initially evaluated at a regional hospital, where routine blood investigations and an MRI spine had been ordered for workup. Subsequently, he had been referred to our oncology center for further workup and management. The patient reported to our center with blood and MRI investigation reports. MRI images were not available for review. The MRI report revealed T1Wt iso to hypointensity and T2/STIR hyperintensity involving the body of D10 vertebra. There were other smaller, ill-defined T2Wt iso to mildly hyperintense and STIR hyperintense lesions in multiple cervical, dorsal, lumbar, and sacral vertebrae. Findings were in favor of neoplastic aetiology with differentials of metastatic disease and bone marrow infiltrative disorder. Subsequently, serum protein electrophoresis with bone marrow aspiration and biopsy were advised for further workup at our oncology center. Blood work and myeloma panel investigations results are presented in Table 1.

**Table 1:** Blood work and myeloma panel investigations

Investigation	Result	Reference range
Hemoglobin (g/dL)	11.9	11.5 - 16.5
Serum creatinine (mg/dL)	3.77	0.5 - 1.5
Serum calcium (mg/dL)	13.3	8.6 - 10.3
Serum Sodium (mEq/L)	140.5	135 - 145
Serum Potassium (mEq/L)	6.23	3.5 - 5.5
Lactate dehydrogenase (U/L)	453	140 - 280
Total protein (g/dL)	10.6	6.4 - 8.3
Albumin (g/dL)	3.98	3.5 - 5.5
Globulin (g/dL)	6.62	2.0 - 3.5
Albumin/globulin ratio	0.6	1.1 - 2.5
Beta 2 microglobulin (mg/L)	6.4	0.8 - 2.5
M band (g/dL)	4.23	
ImmunoglobulinG (g/L)	49.2	6.0 - 16.0
Free kappa (mg/L)	1362.42	3.3 - 19.4
Free lambda (mg/L)	19.67	5.7 - 26.3
K/L ratio	69.26	0.26 - 1.65

Bone marrow aspiration and biopsy were also ordered, revealing 44% plasma cells and 2% blasts. A final diagnosis of IgG kappa type MM was established as International Staging System (ISS) stage III. Subsequently, 18F-FDG PET/CT and 68Ga-CXCR4 PET/CT were also ordered to assess the burden of disease and radiological staging. Both PET/CT scans were performed within an interval of 10 days after obtaining informed consent from the patient. PET/CT imaging was performed 60 minutes after radiotracer injection for both PET agents. Doses of 370 MBq and 200 MBq were used for 18F-FDG and 68Ga-CXCR4, respectively. The results of these PET/CT scans were reviewed by two independent nuclear medicine physicians. Both PET/CT scans were assessed for the pattern of radiotracer uptake and characterized as focal uptake, diffuse uptake, or a mixed pattern of focal and diffuse uptake. The total number of abnormal sites of radiotracer uptake was also calculated to assess overall disease burden and staging (Table 2).

**Table 2:** Analysis of 18F-FDG and 68Ga-CXCR4 PET/CT results 18F-FDG: 18F-fluorodeoxyglucose; CXCR4: C-X-C chemokine receptor type 4; PET/CT: positron emission tomography/computed tomography; DSPS: Durie-Salmon Plus

Variables	18F-FDG PET/CT	68Ga-CXCR4 PET/CT
Focal uptake pattern	21	49
Diffuse uptake pattern	10	6
Mixed focal and diffuse uptake pattern	0	0
Total sites	31	55
Staging (DSPS)	III	III

**Figure 1:**
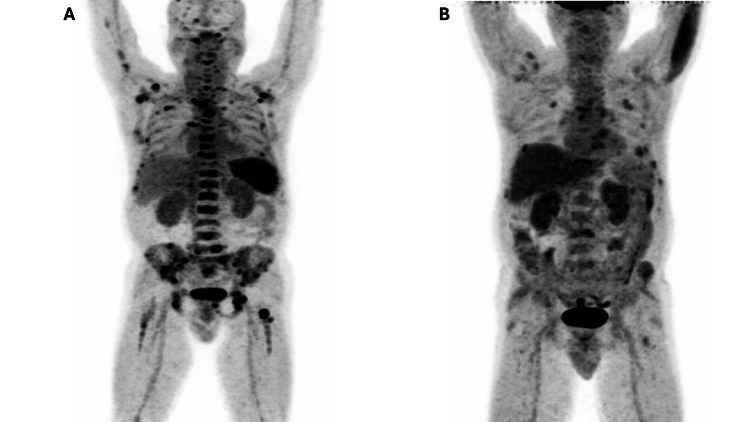
68Ga-CXCR4 and 18F-FDG PET/CT MIP images of the patient (A) MIP 68Ga-CXCR4. (B) MIP 18F-FDG. Image A of 68Ga-CXCR4 PET/CT shows multiple areas of focal increased radiotracer uptake in regions of the axial and appendicular skeleton. In contrast, Image B of 18F-FDG PET/CT shows relatively fewer areas of focal radiotracer uptake. Overall, 68Ga-CXCR4 PET/CT shows a greater burden of disease status in comparison to 18F-FDG PET/CT 18F-fluorodeoxyglucose; CXCR4: C-X-C chemokine receptor type 4; PET/CT: positron emission tomography/computed tomography; MIP: maximal intensity projection

**Figure 2:**
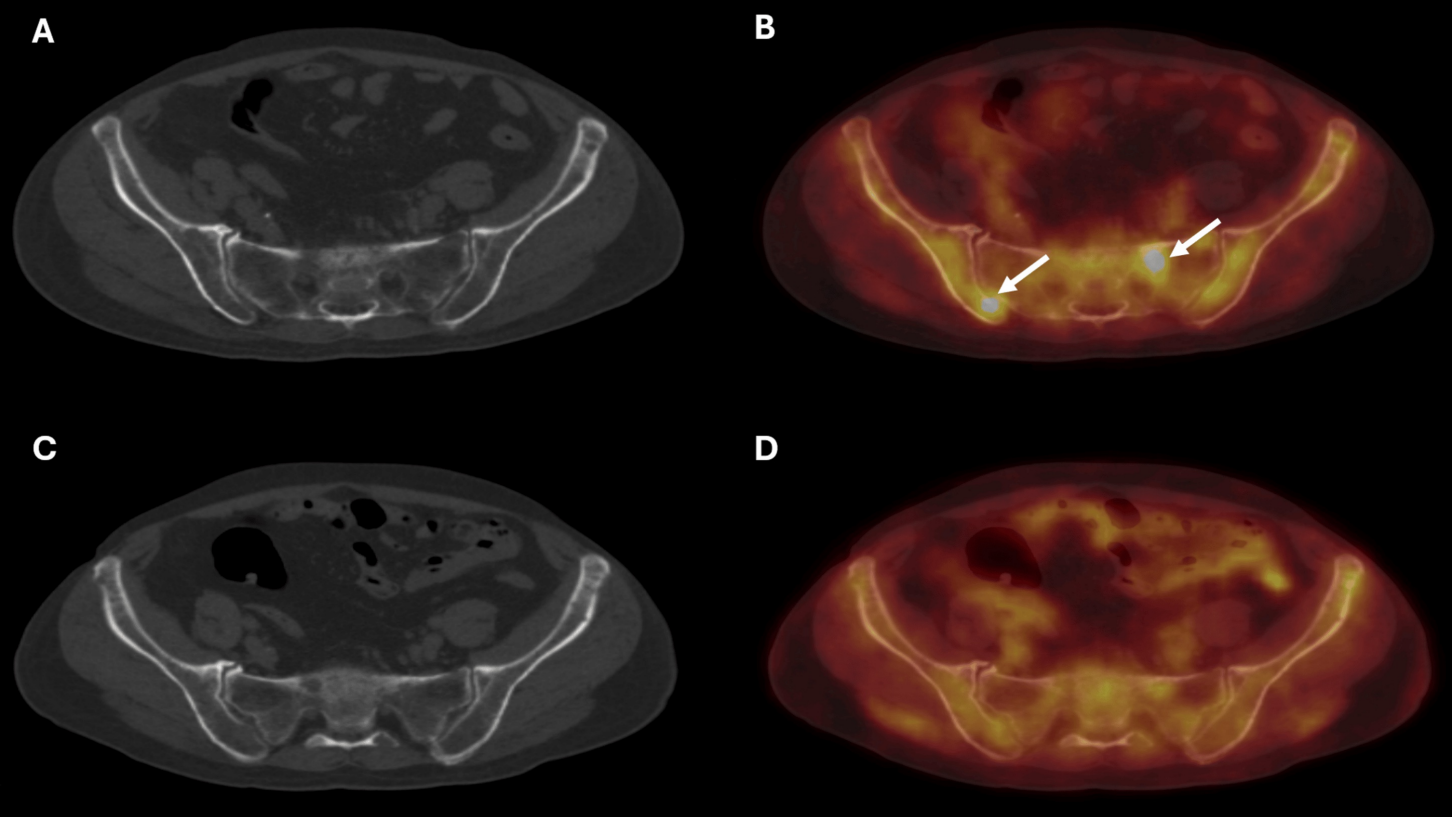
68Ga-CXCR4 and 18F-FDG PET/CT axial images of the patient (A) Axial view of CT. (B) Axial view of 68Ga-CXCR4 PET/CT fusion. (C) Axial view of CT. (D) Axial view of 18F-FDG PET/CT fusion. Image B shows increased 68Ga-CXCR4 uptake in the left ala of the sacrum and right iliac bone (arrows). Image D reveals no focal FDG uptake in the left ala of the sacrum and right iliac bone. Images B and D also show diffuse increased radiotracer uptake involving bilateral iliac bones and sacrum for both the radiotracers. In this example, CXCR4 PET/CT identifies more areas of focal radiotracer uptake CXCR4: C-X-C chemokine receptor type 4; 18F-fluorodeoxyglucose; PET/CT: positron emission tomography/computed tomography

In comparison to 18F-FDG PET /CT, the other modality, 68Ga-CXCR4 PET/CT, identified more sites of radiotracer uptake in the axial and appendicular skeleton (Figure 1). The 68Ga-CXCR4 PET/CT identified a total of 55 lesions in comparison to 18F-FDG PET/CT, which identified 31 lesions. As per the DSPS system, the staging did not change, as both the PET/CT modalities identified more than 20 lesions. The pattern of uptake for both these radiotracers was also analyzed. Focal uptake pattern was seen at 49 sites in 68Ga-CXCR4 PET/CT as compared to 21 sites on 18F-FDG PET/CT. Additional sites of radiotracer uptake were identified in 68Ga-CXCR4 PET/CT (Figure 2).

**Figure 3:**
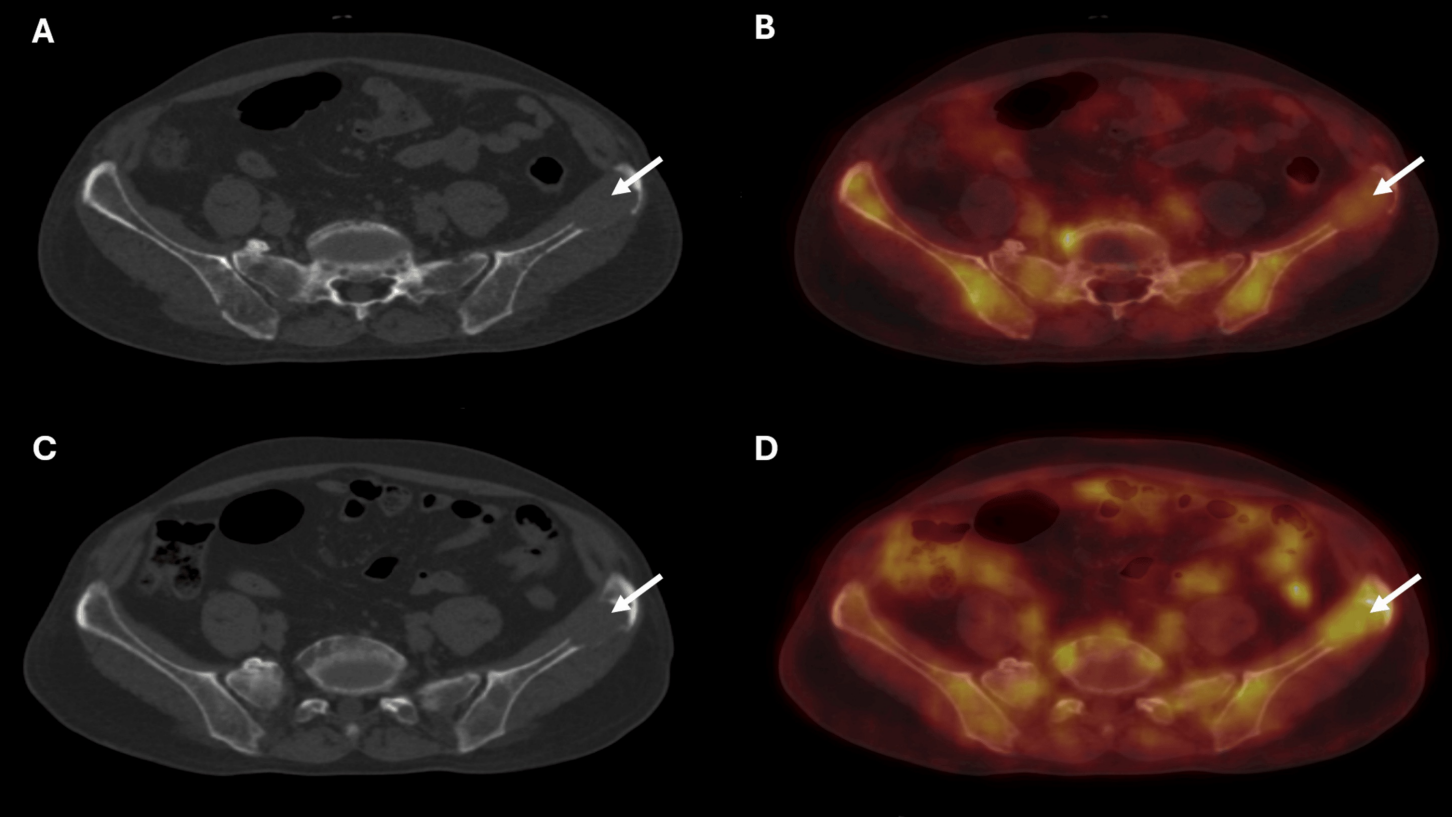
68Ga-CXCR4 and 18F-FDG PET/CT axial images of the patient (A) Axial view of CT. (B) Axial view of 68Ga-CXCR4 PET/CT fusion. (C) Axial view of CT. (D) Axial view of 18F-FDG PET/CT fusion. Images A and B show minimal increased 68Ga-CXCR4 uptake in the lytic lesion with soft tissue component involving the left iliac bone (arrows). Images C and D reveal increased FDG uptake in the lytic lesion with soft tissue component involving the left iliac bone (arrows). This is an example of the lesion showing a higher tumor-to-background ratio in FDG PET/CT 18F-FDG: 18F-fluorodeoxyglucose; CXCR4: C-X-C chemokine receptor type 4; PET/CT: positron emission tomography/computed tomography

Ten sites of diffuse uptake pattern were seen on 18F-FDG PET/CT in comparison to 68Ga-CXCR4 PET/CT, which identified six sites. There were eight sites of diffuse uptake pattern seen on 18F-FDG PET/CT, which were identified as focal uptake patterns on CXCR4 PET/CT. Additionally, 68Ga-CXCR4 PET/CT also identified at least four new sites of diffuse uptake pattern. Hence, 68Ga-CXCR4 depicted a more extensive burden of disease in comparison to 18F-FDG. The maximum tumor-to-background ratio (TBRmax) was calculated as the ratio of the maximum standardized uptake value (SUVmax) of the lesion compared to the background blood pool SUVmax value. The TBRmax values for 68Ga-CXCR4 PET/CT were higher in comparison to those of 18F-FDG PET/CT (Table 3). One exception of a lower TBRmax value in 68Ga-CXCR4 PET/CT was noted in the left iliac bone lesion, which had an associated soft tissue component on CT morphology (Figure 3). Hence, in this MM case, these overall results make 68Ga-CXCR4 a superior PET/CT modality in comparison to 18F-FDG.

**Table 3:** SUVmax and TBRmax values of representative lesions on 18F-FDG and 68Ga-CXCR4 PET/CT SUVmax: maximum standardized uptake value; TBRmax: maximum tumor-to-background ratio; 18F-FDG: 18F-fluorodeoxyglucose; CXCR4: C-X-C chemokine receptor type 4; PET/CT: positron emission tomography/computed tomography

Site	SUVmax FDG	SUVmax CXCR4	TBRmax FDG	TBRmax CXCR4
Blood pool	2.7	3.4	-	-
Right scapula	2.9	8.8	1.07	2.58
Manubrium	2.8	6.1	1.03	1.79
Left femoral neck	3.4	21.1	1.25	6.2
Sacrum	3.0	11.3	1.11	3.32
Left iliac bone	3.7	2.8	1.37	0.82

The patient has been offered VRD (bortezomib + lenalidomide + dexamethasone) induction chemotherapy treatment with the option of stem cell transplant in the future.

## Discussion

Conventional X-ray skeletal survey in patients with MM has been replaced by MRI and PET/CT imaging modalities. 18F-FDG PET is now a commonly used established modality with a proven role in patients with MM [9]. It is not only used in the diagnosis and staging of MM but also in the assessment of response to therapy. 18F-FDG PET, despite being a standard modality, is still not a malignancy-specific agent, and FDG uptake can also be seen in infective and inflammatory conditions, causing false-positive results [10]. To overcome this limitation of 18F-FDG PET, there has been extensive research for the development of malignancy-specific PET radiotracers. One such agent is Pentixafor, which targets protein receptor CXCR4 expressed on plasma cell membranes of immune cells like plasma cells, T cells, B cells, and stem cells. It is overexpressed in hematological malignancies, breast cancer, colorectal cancer, esophageal cancer, head and neck cancers, renal cancer, lung cancer, and gynecological cancers [4,11].

CXCR4 overexpression is associated with unfavorable prognosis and poor overall survival [11]. CXCR4 is also expressed in aldosterone and cortisol-producing functional adrenal adenomas, and hence 68Ga-Pentixafor PET/CT has been used in non-invasive functional imaging of adrenal adenomas [12]. 68Ga-PET radiotracers can be synthesized anytime on the PET/CT site, as these are produced by 68Ge/68Ga generators. This is a logistical advantage, and scheduling 68Ga-based PET scans can be flexible at any time of the day. In contrast, 18F0-FDG is a medical cyclotron-produced and is supply-dependent; the scans need to be scheduled in accordance with to supply chain. 68Ga-Pentixafor PET has been used for imaging in various cancers, including MM. Initial research in MM cases suggests promising results for 68Ga-Pentixafor PET in comparison to 18F-FDG PET.

Lapa et al. compared 68Ga-Pentixafor PET/CT to 18F-FDG PET/CT and laboratory investigations in 35 patients with MM [13]. In this study, 68Ga-Pentixafor PET was found to be superior to 18F-FDG PET in 63% of cases; 16% of the cases were negative on 68Ga-Pentixafor PET but positive on 18F-FDG PET. They concluded that CXCR4 expression frequently occurs in advanced MM, and 68Ga-Pentixafor PET should be a selection tool for CXCR4-directed therapies and prognostic stratification of MM, rather than a diagnostic modality. In another study, Shekhawat et al. compared 68Ga-Pentixafor PET/CT with 18F-FDG PET/CT for staging of MM [14]. In this study, 68Ga-Pentixafor reported a higher disease burden in 68% of patients in comparison to 18F-FDG PET/CT. The authors emphasized that 68Ga-Pentixafor PET/CT should be used in conjunction with 18F-FDG PET/CT and guide CXCR4-based therapies.

In the present study, we also found 68Ga-Pentixafor to be a superior imaging modality in comparison to 18F-FDG, as it revealed a higher disease burden, though staging was not changed in either of these scans. Also, the tumor-to-background ratio (TBRmax) was higher for 68Ga-Pentixafor PET, which makes the interpretation of the scan more objective with possibly lesser inter- and intraobserver variations. A larger number of MM patients need to be assessed to ascertain if 68Ga-Pentixafor can be used as a standalone PET radiotracer for MM, or if it will serve as a tool for patient selection for CXCR4-targeted therapies.

## Conclusions

18F-FDG PET/CT has a proven role and is a widely used modality for the staging of MM. The 68Ga-Pentixafor PET radiotracer is a promising imaging modality for the detection and staging of various malignancies. Initial research suggests favorable results for 68Ga-Pentixafor PET/CT in the staging of patients with MM. It demonstrates a greater extent of disease compared with 18F-FDG PET/CT in advanced cases of MM. 68Ga-Pentixafor PET/CT may be a valuable modality for assessing and guiding CXCR4-targeted therapies in MM, and more research is needed to better define its role in MM. Future research should evaluate the potential role of 68Ga-Pentixafor as a screening modality to guide therapy in patients with MM.
